# A Rare Case of Renal Sarcoidosis

**DOI:** 10.7759/cureus.15494

**Published:** 2021-06-07

**Authors:** Zeeshan Zia, Qasim Z Iqbal, Raffaele A Ruggiero, Sami Pervaiz, Michel Chalhoub

**Affiliations:** 1 Internal Medicine, Northwell Health, New York, USA; 2 Pulmonary and Critical Care, Northwell Health, Staten Island, USA; 3 Northwell Hofstra School of Medicine at Staten Island University Hospital, Northwell Health, Staten Island, USA

**Keywords:** renal sarcoidosis, renal failure, extra pulmonary manifestations of sarcoidosis, chronic granulomatous inflammatory disorder, internal medicine

## Abstract

Sarcoidosis is a multisystem granulomatous disorder characterized by non-caseating granulomas in multiple organs. It most commonly involves lungs and it is very rare to find isolated cases affecting other organ systems with no associated pulmonary findings. We hereby present a case of a young 30-year-old male who was referred to the hospital by his primary medical doctor due to right eye pain secondary to iritis and acute kidney injury (AKI). His initial laboratory studies revealed anemia, AKI, mild hypercalcemia, and the urinary analysis revealed proteinuria. Imaging studies were negative and a kidney biopsy was performed and showed results from the biopsy that revealed diffuse tubulointerstitial disease with early fibrosis, widespread moderate inflammation, multifocal tubulitis, and focal aggregate of epithelioid cells suggestive of granuloma consistent with sarcoidosis. The patient was treated with prednisone. Renal involvement of sarcoidosis is extremely rare (around 0.7%). It has a wide spectrum of presentation including abnormal calcium metabolism, nephrolithiasis, nephrocalcinosis, and acute tubulointerstitial nephritis with or without granulomas. This is a unique case as it shows renal sarcoidosis without coexisting pulmonary finding of hilar lymphadenopathy on chest X-ray. There are very few reported cases of renal sarcoidosis in the literature and this case can add to the pool of those cases. It also emphasizes the need for urgent renal biopsy in the settings of AKI associated with mild to moderate proteinuria. Lack of availability of comprehensive research on the disease may lead to misdiagnosis and delay in treatment.

## Introduction

Sarcoidosis is a chronic granulomatous inflammatory disorder that has an effect on various systems of the body. Amongst the organs damaged by this inflammatory disorder, the lungs are the most commonly affected. Kidneys even though protected from it, can be damaged by sarcoidosis in very rare cases. Renal sarcoidosis hence is not very often seen in routine clinical practice. The presentation of renal sarcoidosis can be vague and can vary according to the underlying pathological mechanism. The diagnosis considering its rarity can be very challenging at times. We were lucky enough to encounter an interesting case of renal sarcoidosis in a young man and we present that case here.

## Case presentation

A 30-year-old male with no past medical history was instructed by his nephrologist to present to the emergency department (ED). However, before the presentation here, the patient went to his primary care physician with a complaint of chronic dry intermittent cough for the last four weeks and pain in his right eye associated with changes in his vision. His outpatient labs revealed that the patient had high levels of serum calcium as well as an acute kidney injury (AKI). He was hence referred to an ophthalmologist and a nephrologist by his primary care physician. The ophthalmologist diagnosed him with iritis, which was prominent in his right eye and progressed to his left eye. His outpatient workup by the nephrologist revealed high calcium and low parathyroid hormone. The laboratory testing for multiple myeloma, HIV, and hepatitis all came back negative. Furthermore, several imaging studies were performed to screen for a malignancy considering high calcium in the setting of low parathyroid hormone. An ultrasound of the abdomen and bladder were negative for any masses; the CT scans of the chest, abdomen, and pelvis performed were negative for any masses or lymphadenopathy. Considering all these findings, the patient was instructed to go to the ED for an elective kidney biopsy. On initial presentation to the ED, he endorsed being completely asymptomatic and his vital signs were within normal limits. His initial laboratory studies (Table 1) were significant for AKI and mild hypercalcemia, whereas the urinary analysis revealed proteinuria (100 mg/dL) (Table 2). Sarcoidosis was high on the list of differential diagnoses due to the combination of iritis, chronic dry cough, and laboratory findings. The nephrology and pulmonology teams consulted in the hospital both recommended a kidney biopsy. We also sent in laboratory studies for other possible causes of these symptoms such as systemic lupus erythematosus or scleroderma. The following day a kidney biopsy was performed. The 25-hydroxy vitamin D levels were found to be low. Results for antinuclear antibody (ANA), antineutrophil cytoplasmic antibody (ANCA), complement, thyroid stimulating hormone (TSH), angiotensin-converting enzyme (ACE) were all found to be within normal limits. The ferritin level was high in the setting of anemia, which suggested the presence of chronic disease. On hospital day 2, the preliminary results from the biopsy revealed diffuse tubulointerstitial disease with early fibrosis, widespread moderate inflammation, multifocal tubulitis, and focal aggregate of epithelioid cells suggestive of granuloma. These findings were all consistent with a high suspicion for sarcoidosis. The patient was subsequently started on a prednisone taper. He was discharged and then referred to follow up with a multidisciplinary team for long-term care involving nephrology, pulmonology, and hematology. A few days later the official report of the kidney biopsy confirmed sarcoidosis.

**Table 1:** Laboratory work-up. AST, aspartate aminotransferase; ALT, alanine aminotransferase; eGFR, estimated glomerular filtration rate

Sodium	140
Potassium	4.3
Chloride	104
Carbon dioxide	23
Anion gap	13
Blood urea nitrogen	35
Creatinine	3.7
Calcium	11.1
Protein, total	8
Albumin	4.7
Alkaline phosphatase	114
AST	18
ALT	43
eGFR	21

**Table 2:** Urine analysis.

Color	Light yellow
Urine appearance	Clear
Bilirubin	Negative
Ketone	Negative
Specific gravity	1.013
Protein	100 mg/dL
Urobilinogen	<2 mg/dL
Nitrate	Negative
Leukocyte esterase concentration	Negative
Blood	Small
Epithelial cells	3
Red blood cells	5
Bacteria	Negative
White blood cell	6
Hyaline casts	0
Glucose qualitative urine	200 mg/dL

## Discussion

Sarcoidosis was first described in 1877 as a multisystem granulomatous disorder of unknown etiology. It is characterized pathologically by the presence of noncaseating granulomas in involved organs, most commonly involving the lungs and intrathoracic lymph nodes [1]. It usually affects young adults (80% of cases occur between adults age 20 and 50 years), and initially usually present with one or more of the following abnormalities; bilateral hilar adenopathy, pulmonary reticular opacities, skin, joint, and/or eye lesions. It is more prevalent in African American and women populations [2].

Renal involvement of sarcoidosis is extremely rare (around 0.7%). It has a wide spectrum of presentation including abnormal calcium metabolism, nephrolithiasis, nephrocalcinosis, and acute tubulointerstitial nephritis with or without granulomas [3-4]. Non-granulomatous tubulointerstitial nephritis is the most common histological entity (44%), followed by granulomatous interstitial nephritis (GIN, 30%), IgA-GN (26%), and nephrocalcinosis (11%) [5]. Some of the rare manifestations include Amyloid A (AA) amyloidosis, urinary tract obstruction with renal masses, and glomerular diseases such as membranous nephropathy, Immunoglobulin A (IgA) nephropathy, minimal change disease, proliferative or crescentic glomerulonephritis, and focal segmental glomerulosclerosis [6-8]. The presentation of renal sarcoidosis would vary according to the underlying pathology. Sarcoid-related interstitial nephritis is usually identified as the initial presentation of sarcoidosis and rarely develops among patients who have a longstanding diagnosis of sarcoidosis [9].

Most patients with interstitial nephritis present with elevated creatinine that is detected on routine screening or as part of their initial evaluation for sarcoidosis. In the study [9] amongst 47 patients with sarcoidosis-related interstitial nephritis, 46 presented with an elevated creatinine on diagnosis. In the study, 66% of patients presented with moderate proteinuria and glomerular interstitial nephritis. In another study of 27 patients, non-granulomatous interstitial nephritis was more common when compared to GIN [5]. This is backed by the observation that the likelihood of GIN in the biopsy increases with deteriorating renal function where the burden of granulomas is high because of advanced disease [2]. Some patients can present with chronic kidney disease (CKD) (29 out of 47). The majority of the patients in the above-mentioned study [9] had diffuse active sarcoidosis. The usual presentation would include fatigue and typically b-grade symptoms. This was seen in a study where systemic symptoms, including fatigue, weight loss, and fever, were present in 20 patients (42%) [10]. Systematic investigations and larger cohorts (n > 50) regarding the frequency of renal sarcoidosis are scarce and those present show substantial variability in a frequency ranging from less than 1% to 30%-50% of all patients with sarcoidosis [2, 11]. Some evidence would include a case report from 1993 which describes a young man with a known history of pulmonary sarcoidosis presenting with acute renal failure (ARF) and hypercalcemia; the renal biopsy was positive for calcium deposits in cortex and medulla, interstitial fibrosis, and a single non-caseating granuloma in the medulla [6]. Literature regarding it would further include a 46-year-old female who presented with cough, AKI, and bilateral anterior uveitis. Renal biopsy revealed acute interstitial nephritis with non-caseating granulomas, giant cells, lymphocytic infiltration, and rare calcium deposits on light microscopy [4]. Furthermore, an old review of the literature from 1986 described 22 cases of renal sarcoidosis all presenting with different systemic presentation and biopsy findings. Amongst the 22 patients, three failed to respond to treatment eventually dying from acute renal failure and relapse occurred in four of the patients. Importantly though no relapse was reported later than nine months after initiation of treatment with a corticosteroid. The majority of patients who did respond to treatment had residual renal impairment after up to 30 months of steroid treatment [10]. There is also a case series from Germany describing six patients with renal involvement in sarcoidosis without any other systemic findings [12]. The six patients were treated with corticosteroids and serum creatinine levels decreased significantly in four of the patients (>50% decrease). Therefore, even in the absence of other organ manifestations, sarcoidosis can be the cause of renal insufficiency, and it responds well to corticosteroid treatment.

The literature demonstrates the high importance of kidney biopsy while evaluating unexplained deterioration of renal function in patients. The suspicion for diagnosis should be high in patients presenting with elevated creatinine associated with systemic findings like cough, fever, fatigue, or weight loss. While the renal biopsy is diagnostic, the use of serum angiotensin-converting enzyme (ACE) level is debatable. This was also seen in a study, where only one out of five patients with isolated sarcoid granulomatous interstitial nephritis had elevated ACE levels [13]. The baseline diagnostic workup to establish the diagnosis of renal sarcoidosis further includes the assessment of renal function by serum creatinine and calculation of eGFR, the quantification of total protein secretion in the urine and urine sediment, serum calcium, urine calcium excretion in 24 hours, serum 1,25-Vitamin D, and an ultrasound of the kidneys. Differential diagnosis would include drug-induced interstitial nephritis, infections like tuberculosis and other mycobacterium infections, GPA, brucellosis, histoplasmosis, and autoimmune diseases like tubulointerstitial nephritis with uveitis (TINU) syndrome, Wegener granulomatosis, and rarely, Crohn’s disease [14-15]. Sarcoidosis and medications are the most common causes of renal granulomatosis. In a study done in France in 2007 [14] on 40 patients with renal granulomatosis, the following etiologies were found; sarcoidosis in 20 patients, drug-induced in seven patients, tuberculosis in three patients, Wegener granulomatosis in two patients, leprosy in one patient, *Mycobacterium avium* infection in one patient, Crohn’s disease in one patient, and no etiology identified in five patients. This was also seen in a study from Harvard University which identified 46 cases of GIN and concluded that three-quarters of the cases were either drug-induced or due to sarcoidosis [15].

Glucocorticoids are the treatment of choice but the recovery rate is decreased in longstanding disease with irreversible renal damage. The response to glucocorticoids was best noted in a study of 47 patients with renal sarcoidosis [9] mentioned above. The patients received steroids and at a median follow-up of 24 months, complete and partial response occurred in 30 and five patients, respectively. No response was reported in those with greater than 50% fibrosis by histologic examination. Importantly, the presence of hypercalcemia on presentation correlated with a complete response to glucocorticoid therapy at one year. In patients with sarcoidosis-related interstitial nephritis who cannot tolerate or do not respond to glucocorticoids, alternative therapies that are effective in pulmonary sarcoidosis such as methotrexate, chloroquine, azathioprine, a combination of these drugs, or, as a last resort, tumor necrosis factor antagonists or their biosimilars may be tried [16-17].

## Conclusions

Renal sarcoidosis is an underestimated cause of severe morbidity in the young population. Lack of availability of comprehensive research on the disease may lead to misdiagnosis and delay in treatment. Suspicion should be high especially for young patients presenting with AKI associated with mild to moderate proteinuria with or without systemic involvement. An early renal biopsy should be performed after ruling out other causes and treatment should be started aggressively to avoid long-term morbidity and reduce mortality.
